# Distance from forest edge affects bee pollinators in oilseed rape fields

**DOI:** 10.1002/ece3.924

**Published:** 2014-01-15

**Authors:** Samantha Bailey, Fabrice Requier, Benoît Nusillard, Stuart P M Roberts, Simon G Potts, Christophe Bouget

**Affiliations:** 1 National Research Institute Sciences & Technologies Environment & Agriculture Irstea, Res Unit Biodiversity45290, Nogent-sur-Vernisson, France; 2 UE Entomologie, INRA, UE 1255F-17700, Surgères, France; 3 Centre d'Etudes Biologiques de Chizé, CNRS, UPR 193479360, Beauvoir sur Niort, France; 4 Centre for Agri-Environmental Research (CAER), University of ReadingReading, U.K

**Keywords:** *Andrena*, bee dispersal, ecosystem service, foraging range, *Nomada*, partial habitats, wild bees

## Abstract

Wild pollinators have been shown to enhance the pollination of *Brassica napus* (oilseed rape) and thus increase its market value. Several studies have previously shown that pollination services are greater in crops adjoining forest patches or other seminatural habitats than in crops completely surrounded by other crops. In this study, we investigated the specific importance of forest edges in providing potential pollinators in *B. napus* fields in two areas in France. Bees were caught with yellow pan traps at increasing distances from both warm and cold forest edges into *B. napus* fields during the blooming period. A total of 4594 individual bees, representing six families and 83 taxa, were collected. We found that both bee abundance and taxa richness were negatively affected by the distance from forest edge. However, responses varied between bee groups and edge orientations. The ITD (Inter-Tegular distance) of the species, a good proxy for bee foraging range, seems to limit how far the bees can travel from the forest edge. We found a greater abundance of cuckoo bees (*Nomada* spp.) of *Andrena spp*. and *Andrena spp*. males at forest edges, which we assume indicate suitable nesting sites, or at least mating sites, for some abundant *Andrena* species and their parasites (Fig. 1). *Synthesis and Applications*. This study provides one of the first examples in temperate ecosystems of how forest edges may actually act as a reservoir of potential pollinators and directly benefit agricultural crops by providing nesting or mating sites for important early spring pollinators. Policy-makers and land managers should take forest edges into account and encourage their protection in the agricultural matrix to promote wild bees and their pollination services.

## Introduction

Pollinators play an important functional role in most terrestrial ecosystems and provide a key ecosystem service (Ashman et al. 2004). Insects, particularly bees, are the primary pollinators for the majority of the world's angiosperms (Ollerton et al. 2012). Without this service, many interconnected species and processes functioning within both wild and agricultural ecosystems could collapse (Kearns et al. 1998). *Brassica napus* (oilseed rape, OSR) represents the most widespread entomophilous crop in France with almost 1.5 Mha in 2010 (FAOSTAT August 10th, 2012). Results differ between varieties, but even though it seems that OSR produces 70% of its fruits through self-pollination (Downey et al. 1970 in Mesquida and Renard 1981), native bees are also known to contribute to its pollination (Morandin and Winston 2005; Jauker et al. 2012). Bee pollination leads to improved yields (Steffan-Dewenter 2003b; Sabbahi et al. 2005) and to a shorter blooming period (Sabbahi et al. 2006), thus increasing the crop's market value (Bommarco et al. 2012). The most widely used species in crop pollination is the honeybee (*Apis mellifera* L) which is sometimes assumed to be sufficient for worldwide crop pollination (Aebi and Neumann 2011). However, this assertion has been questioned by different authors (Ollerton et al. 2012), and several studies show that many wild bees are also efficient pollinators of crops (Klein et al. 2007; Winfree et al. 2008; Breeze et al. 2011). Recently, Garibaldi et al. (2013) found positive associations of fruit set with wild-insect visits to flowers in 41 crop systems worldwide. They demonstrate that honeybees do not maximize pollination, nor can they fully replace the contributions of diverse, wild-insect assemblages to fruit set for a broad range of crops and agricultural practices on all continents with farmland. Unfortunately, not only are honey bees declining due to a variety of different causes (vanEngelsdorp et al. 2009), wild bee populations are also dwindling (Potts et al. 2010). Their decline has been documented in two Western European countries (Britain and the Netherlands) by comparing data obtained before and after 1980 (Biesmeijer et al. 2006). These losses have mostly been attributed to the use of agrochemicals, the increase in monocultures, the loss of seminatural habitat and deforestation (Steffan-Dewenter et al. 2002; Steffan-Dewenter and Westphal 2008; Brittain and Potts 2011).

**Figure 1:**
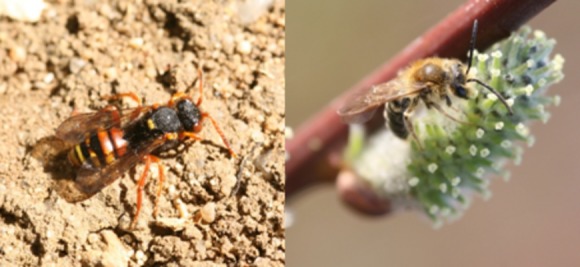
Left, a *Nomada sp* male; right, an *Andrena sp* male.

Several studies have shown the importance of natural or seminatural habitats in sustaining pollinator populations or pollination services close to fruit crops (Steffan-Dewenter 2003a; Kremen et al. 2004; Greenleaf and Kremen 2006a; Carvalheiro et al. 2010). Morandin and Winston (2006) presented a cost–benefit model that estimates profit in OSR agroecosystems with different proportions of uncultivated land. They calculated that yield and profit could be maximized with 30% of the land left uncultivated within 750 m of field edges. Other studies have demonstrated a negative impact of the distance from forests on pollination services or bee abundance and richness both in tropical ecosystems (De Marco and Coelho 2004; Blanche et al. 2006; Chacoff and Aizen 2006) and in temperate ecosystems (Hawkins 1965; Taki et al. 2007; Arthur et al. 2010; Watson et al. 2011).

These studies all suggest that natural or seminatural habitats are important sources of pollinators, probably because they provide “partial habitats” (Westrich 1996) such as complementary mating, foraging, nesting, and nesting materials sites that bees need to complete their life cycle. In this study, we focused on the effect of distance to forest edge on bee assemblages in OSR ecosystems. Forest edges could provide one or more important partial habitats for different bee species in agricultural landscapes, in particular when associated with a mass-flowering crop such as OSR (Le Feon et al. 2011). For example, the availability of untilled soil and dead branches might provide ground-nesting and cavity-nesting bee species with numerous nesting sites. Moreover, during spring at least, the understory and the forest edge can provide cover containing flowering plants and wild trees such as *Prunus spp*, *Castanea sativa,* or *Salix spp* and thereby allow bees to find alternative floral resources.

During spring 2010 and 2011, in two areas in France, we examined wild bee abundance and taxa richness both along forest edges and inside OSR fields at different distances from the forest. Like other taxa, bees respond to environmental variables according to their biologic traits that determine access and requirements for nesting, mating, and forage resources, species mobility or physiological tolerance. Specifically, we hypothesized that (1) bee abundance, species richness, and composition of bee communities within the crop field are dependent on the distance from the forest edge (where complementary floral resources, nesting sites, shelters, etc. can be found) and on the orientation of the forest edge; (2) the identity of bees in the crop is related to their foraging range which we measured with the ITD (Inter-Tegular distance); (3) the forest edge may be the nesting or mating sites for cavity-nesting or ground-nesting bees such as *Osmia spp* or *Andrena spp* which are important groups of potential early spring pollinators for OSR.

## Materials and Methods

### Study sites

**Figure 2:**
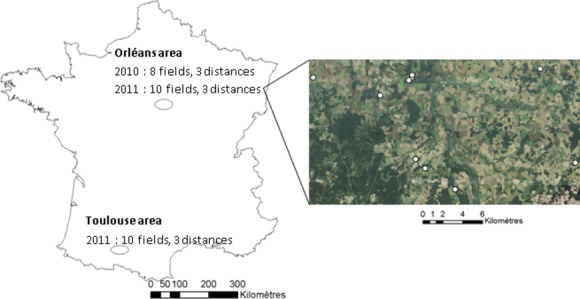
Location of study areas and spatial arrangement of our sampling design.

**Figure 3:**
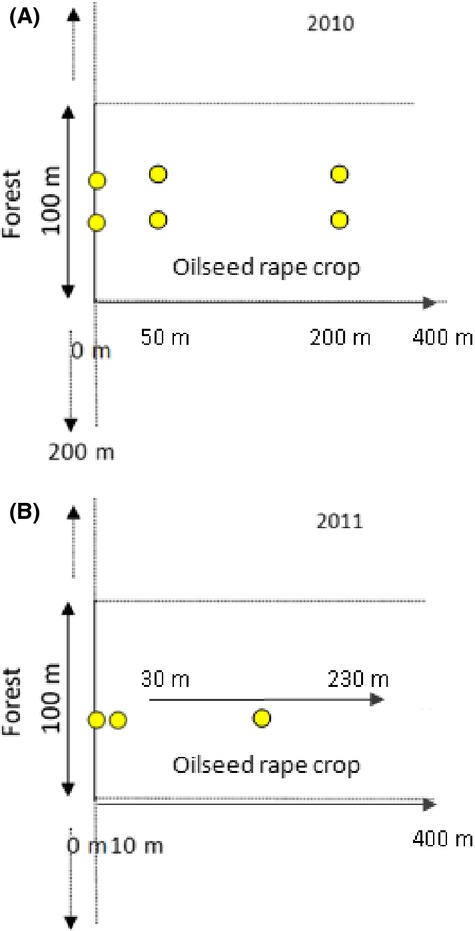
Design used to survey bees in oilseed rape crops at different distances from the forest edge. Circles represent yellow pan traps.

The field work was conducted in 2010 near Orleans, France (latitude 47.8537191, longitude 2.7499075), and in 2011 in the same area and in addition, near Toulouse, France (latitude 43.3030938, longitude 0.9914780). These two study areas are 700 km from each other. In 2010, we selected eight fields sown with *B. napus* and in 2011, a total of ten fields in both areas (Fig. 2). The 28 fields were selected with at least one of their sides directly adjacent to a forest with indigenous deciduous tree species (mainly *Quercus*, *Carpinus* and *Populus* spp.). We classified 11 forest edges as “cold orientation” (northern and eastern exposure) and 17 forest edges as “warm orientation” (southern and western exposure) according to the amount of Celsius degree they received during the day. The fields we selected in 2010 and 2011 had forest edges of at least 100 m in length. In 2010, we had two study point distances from forest edge, 50 m and 200 m. Our 200-m study points were distant from other edges by at least 200 m. In 2011, we also had two study point distances from forest edge, 10 m and the further one varied from 30 to 230 m (Fig. 3).

### Bee sampling

We used yellow pan traps to sample bees, while the OSR was in bloom; this is a common passive sampling method (Dafni et al. 2005 in Westphal et al. 2008). The traps were plastic bowls (approximately 30 cm in diameter and 23 cm in height) with an UV-reflecting paint (S.P.R.L, Spray-color 18 133UK, Brussels, Belgium) sprayed on the inside. They were mounted on wooden poles at vegetation height (Westphal et al. 2008) and filled with approximately 2.4 L of water, 0.6 L of monopropylene glycol for conservation, and a few drops of liquid soap to lower surface tension, and then were exposed for 15 days during the blooming period. In 2010, we placed two pan traps at each distance from forest edge: 0 m (forest edge), 50 m, and 200 m into the crop. In 2011, we placed one pan trap at each distance: 0 m (forest edge), 10 m, and a third location varying between 30 and 230 m into the crop. Collected specimens were stored in a freezer, then dried, mounted, and identified to the species level when possible. Some specimens could only be determined to the genus (*Nomada, Sphecodes*) or subgenus (*Micrandrena*) level. The specimens were also separated into males and females.

### Data analysis

Hypothesis 1: bee abundance, species richness, and composition of bee communities within the crop field are dependent on the distance from the forest edge and forest edge orientation

We constructed generalized additive mixed models (R; mgcv package) to test our hypotheses about total bee abundance and bee species richness as a function of distance and orientation (2-level categorical variable specifying a cold or warm orientation). In addition to the interaction between distance and orientation, we included year (*n* = 2) and field area (*n* = 2) as additional fixed effects and the field identity (*n* = 28) as a random effect. Residuals analyses motivated us to use a Poisson distribution for the abundance and a normal distribution for the species richness (Table 1). In our analysis of species richness, we also included total abundance as a covariate.

**Table 1:** Estimates (± SE) of ecological effects from generalized additive mixed-effect models for bee abundance, species richness, mean female ITD, *Andrena* females and males, and *Nomada*.

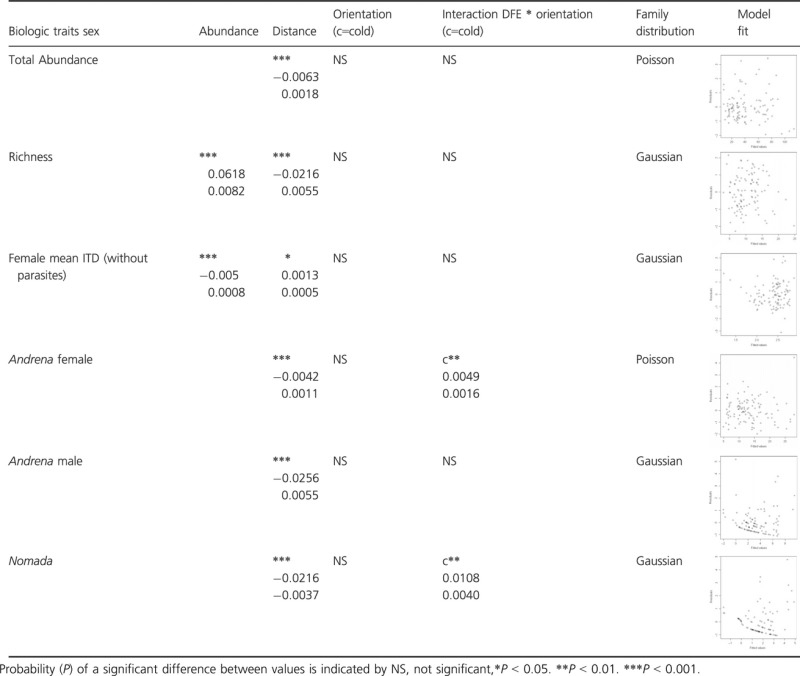

To examine how the composition of the bee community varied with distance and orientation, we used canonical analysis of principal coordinates (CAP) (R package: vegan, function: capscale; R Core Team 2012). This method allowed us to quantify and test the individual contribution of qualitative variables (year, geographic area, field, and orientation) and the quantitative variable (distance) to variations in total assemblage composition. We used the Jaccard similarity index and carried out an inertia partitioning to estimate the total variance in assemblage composition, total constrained inertia (i.e., explained by all the variables included in the model), and the relative individual contribution of each variable to the constrained inertia (Anderson and Willis 2003).

Hypothesis 2: the identity of bees in the crop is related to their foraging range

To examine how bee identity varied with distance and orientation of forest edge, we examined how the mean female ITD (Inter-Tegular distance: the distance between the bases of the two wings) varied with distance and orientation. As above, we modeled the mean female ITD using a generalized additive mixed model. In addition to the interaction between distance and orientation, we included year (*n* = 2) and field area (*n* = 2) as additional fixed effects and the field identity (*n* = 28) as a random effect. We used a normal distribution and we also included total abundance as a covariate. Only females and traps with at least two specimens were included in this analysis (2 traps were therefore excluded). Males were not included in the analysis of mean ITD because they do not take care of brood so they do not collect pollen; their principal requirement is finding females with which to mate. On the contrary, females take care of the brood so they must find appropriate nesting sites and supply the larvae with food. Moreover, females exhibit central-place foraging, so they actively travel from crop to nest. They are the actual OSR pollinators. The parasites *Bombus* (*Psithyrus*), *Nomada,* and *Sphecodes* were not included in the analysis of the mean ITD because they also do not take care of their broods; their presence or movements may be more linked to their nest host (Williams et al. 2010).

Hypothesis 3: the forest edge may be the nesting or mating sites for cavity-nesting or ground-nesting bees

To estimate the importance of forest edge for ground-or cavity-nesting bees, we constructed a generalized additive mixed model as above. We focused only on the *Andrena* responses because (1) other groups such as the cavity-nesting bees (*Osmia spp*) were probably underestimated because of the sampling method used (Westphal et al. 2008; Sobek et al. 2009); (2) *Andrena* were the only taxa whose males and parasites had already emerged and could be used as indirect indicators of nesting or mating sites; and (3) other studies in similar areas had already shown that *Andrena* are important visitors to *B. napus* (Delbrassine and Rasmont 1988; Le Feon et al. 2011).

We investigated the response of *Andrena* females and males separately. For *Andrena* females, our model contained the interaction between distance and orientation, and we included year (*n* = 2) and field area (*n* = 2) as additional fixed effects and the field identity (*n* = 28) as a random effect. Residuals analysis suggested a Poisson distribution. For the analysis of *Andrena* males, we further included a factor structuring the variance of error using the “weights” distribution function (varpower). In this case, we used a Gaussian distribution (Table 1).

Finally, for this hypothesis, we also examined the response of the *Andrena* cleptoparasites, *Nomada,* using the same model structure as for *Andrena* males.

## Results

**Figure 4:**
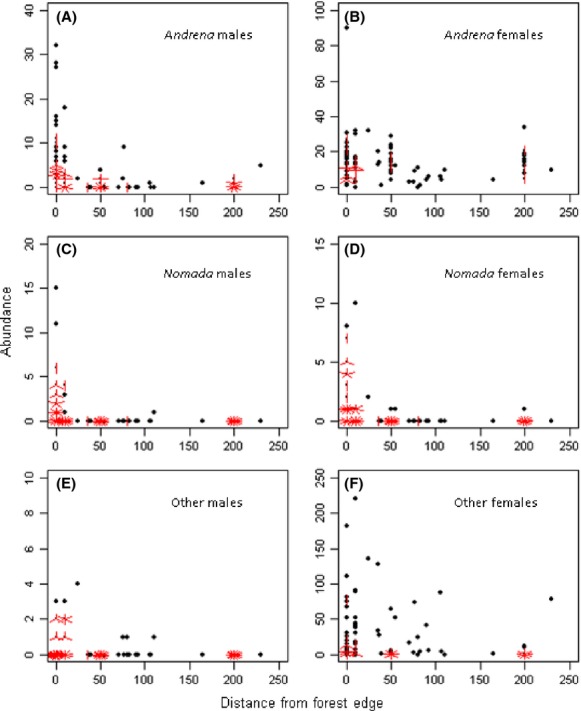
Abundance as a function of distance from the forest edge for different bee groups. We show absence and presence values and use different scales on Y-axes for clarity. Multiple points are plotted as “sunflowers” with multiple leaves (“petals”) such that over-plotting is visualized.

A total of 4594 individuals representing 83 taxa from 6 families, and 12 genera were recorded. The most abundant families were *Halictidae* (49.1% of total abundance, 31 species) and *Andrenidae* (39.5% of total abundance, 36 species). Their parasites, *Sphecodes* (12 specimens) and *Nomada* (191 specimens, 101 females, 90 males), respectively, represented 4.4% of the total abundance. The *Apidae* (*Apis* and *Bombus* spp.) family represented only 5.7% of total abundance. Furthermore, all *Bombus* spp. were queens indicating that colonies had not yet been established at the time of the study. The *Bombus* parasites (*Bombus* (*Psithyrus*) *rupestris, Bombus* (*Psithyrus*) *sylvestris,* and *Bombus* (*Psithyrus*) *vestalis*) with a total of 27 specimens accounted for 0.6% of total abundance. Females for all taxa combined represented 89.1% of total abundance with 4095 specimens, and males only 10.9% (499 specimens) with *Andrena* and *Nomada* males making up, respectively, 74.5% and 18% of male abundance (Fig. 4). *Halictidae* and *Apidae* males emerge later and were therefore absent in our samples.

Hypothesis 1: bee abundance, species richness and composition of bee communities within the crop field are dependent on the distance from the forest edge and forest edge orientation

Distance had a significant negative effect on total abundance and richness (Table 1). The orientation of forest edge and its interaction with distance had no significant effect on total abundance, richness, abundance of *Andrena* males and females mean ITD (Table 1). We observed a positive effect of the interaction between cold orientation and distance on *Andrena* females and *Nomada* abundance. In other words, we observed a decrease in *Andrena* females and *Nomada* abundance with increasing distance from warm edges. Conversely, we observed an increase in *Andrena* females and *Nomada* abundance with increasing distance from cold edges (Data S1). However, for the *Nomada*, the model did not describe the data very well.

Inertia partitioning by CAP (canonical analysis of principal coordinates) showed that distance provided the second largest contribution to the variance in bee assemblages (29.4%), the first explanatory variable being the field ID (48.8%). Distance and field ID were the only significant variables with an independent contribution; the others had only joint contributions (Table 2).

**Table 2:** Results of the canonical analysis of principal coordinates on the bee assemblage for the five factors.

	Total inertia	Pr (>*F*)	% constraint inertia	% own contribution	% joint contribution
Field ID	70.54	0.005	48.8	13.1	86.9
Distance	42.55	0.005	29.4	15.7	84.3
Area	20.29	0.005	14.0	0.0	100.0
Year	8.69	0.005	6.0	0.0	100.0
Orientation	2.56	0.067	1.8	0.0	100.0
Residuals	8.64

Hypothesis 2: the identity of bees in the crop is related to their foraging range

**Figure 5:**
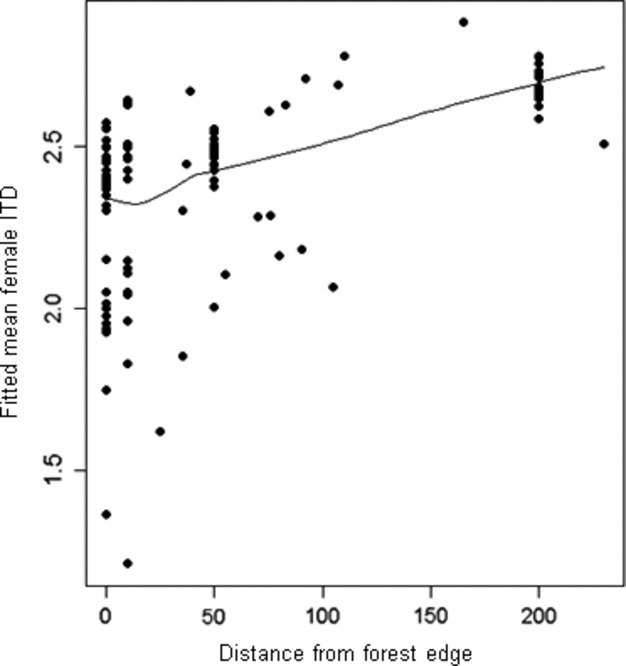
Fitted GAMM model of the response of female mean ITD including distance, year, female abundance, and geographic coordinates as fixed factors and geographic area and field as random factors.

Distance had a significant positive effect on mean female ITD (Fig. 5). In other words, the further away collected bees were from the edge, the larger they were (Table 1).

Hypothesis 3: forest edge as nesting or mating sites for *Andrena*

For the *Andrena*, both females and males were negatively affected by longer distances. *Nomada* were also apparently negatively affected by longer distances; however, the model did not describe the data very well (Table 1). Even so, we decided to retain this model because of the high proportion of *Nomada* present at forest edges (81.7% of their total abundance).

## Discussion

Hypothesis 1: bee abundance, species richness, and composition of bee communities within the crop field are dependent on the distance from the forest edge and forest edge orientation

In our study, we found a negative effect of distance from forest edge on bee abundance and richness. Distance also greatly affected assemblage composition. Our results provide evidence that distance strongly determines the spatial distribution of bees in the OSR field. In a meta-analysis, Ricketts et al. (2008) showed that native pollinator visitation rate drops to 50% of the maximum at a location 668 m away from natural habitats. Some other studies focusing on the effect of forest on bee visits or pollination services are consistent with these results (e.g., Hawkins 1965; De Marco and Coelho 2004; Chacoff and Aizen 2006). Together with ours, these studies highlight that forest edges are likely to be a pollinator source for different crops. Indeed, forest edges present a complex vertical structure and undisturbed soil offering shelter for all bees and a wide range of nesting sites for both cavity-and ground-nesting bee. In addition, they provide a diversity of floral resources throughout the bees' activity period. Finally, these studies also suggest that the pollination of the mass-flowering crop, OSR, could be negatively affected by too great distance from the forest (Morandin and Winston 2005), unless the few species that venture farther afield can provide on their own the supplementary pollination necessary for the crop.

We also observed a positive effect of the interaction between cold orientation and distance on *Andrena* females and *Nomada* abundance. This is consistent with the ecological requirements of solitary bees; they are thermophilous insects so they prefer warm exposed sites for foraging. They may therefore travel further into the field to forage in well-exposed areas. Moreover, rapeseed flowering could be sparse and occurs later along cold forest edges. In that case, bees would probably forage further into the crop where better exposure has encouraged more abundant floral resources.

Hypothesis 2: the identity of bees in the crop is related to their foraging range

In contrast to Lentini et al. (2012), we found that larger female bees were found in the fields further from the forest edge. However, all the fields in Lentini et al.*'s* study contained small untilled areas that could have provided alternative nectar sources or nesting sites and acted as local population sources within the otherwise homogeneous fields. Arthur et al. (2010) also presumed that the absence of an edge effect on solitary bees in OSR might indicate that some bees were nesting inside the crop fields, with minimum tillage technique ground nesting may be possible. In our study, we assumed that: (i) the recorded taxa could not nest in the field itself as mechanical tillage was carried out at least once a year and (ii) some taxa must have covered distances of up to 230 m to reach the OSR field from their nesting sites on the forest edge. We hypothesized that females would be distributed according to their foraging range, calculated by measuring their ITD (Greenleaf et al. 2007). In our study, we found that mean ITD increased with distance from forest edge. Overall, we found that distance was the second most important explanatory factor for the variance in bee communities. For large taxa, the higher energy consumption required to fly further may well be compensated for by less competition for forage resources. The social taxa, *Bombus* spp. and *A. mellifera*, may benefit even more than solitary taxa from the lower competition in the center of the plot because they need to store large amounts of resources to start colonies (Herrmann et al. 2007; Westphal et al. 2009). Additionally, the decline in total bee abundance with increasing distance into the OSR field may reflect a dilution effect: pollinators in the middle of the field have more flowers to choose from away from the forest edge (Arthur et al. 2010). Indeed, even though several species of solitary bees have been found to be able to return from distances of up to 400 meters (Zurbuchen et al. 2010), the smaller species' foraging ranges probably remain rather limited if resources are abundant nearby. This could result in a negative impact on pollination efficiency far from the forest edge with a decrease in interspecific interactions (Greenleaf and Kremen 2006b).

Hypothesis 3: forest edge as nesting or mating sites for *Andrena*

Wild bee nests are difficult to locate in the field (Waters et al. 2011), unless a very limited area is intensively studied. Therefore, we decided to use the distribution patterns of males and nest parasites as general indicators of the areas likely to be used by *Andrena* for nesting or mating; indeed, these two groups' activity is mostly, although not exclusively, focused around nesting or mating sites rather than forage sites (Eickwort and Ginsberg 1980). *Andrena* males patrol areas, marking vegetation with mandibular gland secretions around the nesting sites of females or their food plants (Tengo 1979; Ayasse et al. 2001) or actively search for receptive females at emergence sites (Butler 1965; Tengo 1979). The reproductive success of *Nomada* depends on the capacity of females to find host nests and gain entry into them (Tengo and Bergstrom 1977; Cane 1983). In our study, the preference shown by both *Andrena* males and their cleptoparasites for forest edges indicates suitable nesting, or at least mating sites, for some abundant *Andrena* species and their parasites. This is consistent with Calabuig (2000) who found that the abundance of males and inquilines was significantly higher along forest edges than along several of the other linear habitats tested. Moreover, we observed that several abundant females (*A haemorrhoa, A nitida, A nigroaenea,* and *A cineraria*) occurred at different distances, while their males were most abundant along forest edges. Therefore, forest edges may not just be “partial habitats”; they could be a population sources for potential pollinators to OSR fields.

### Implications for bee conservation and agricultural landscape management

The main objective of this study was to assess whether forest edges are an important partial habitat for potential OSR pollinators. Our results clearly support this assumption. We found that the forest edge is likely to be a nesting site and/or mating site for an important group of pollinating bees, the *Andrenidae*. Furthermore, the forest edge is a potential foraging site for all bees because of the early spring-flowering trees or forbs it contains. Therefore, taking into account, the proportion of forest edges around a field could be an indirect way to measure direct factors such as food availability or the presence of suitable pollinator nesting sites and/or mating sites in a landscape (Roulston and Goodell 2011), at least during spring. We also show that forest edge value may vary depending on microclimatic conditions such as the amount of sunlight it receives. We therefore recommend that future studies include forest edges and seasonality as explanatory variables to explain bee abundance or richness in a given landscape.

The decrease in pollinators with distance seems to be caused by flight costs as indicated by the mean increase in bee size with distance. Therefore, preserving untilled conservation land inside crop fields may be a way to offset the absence of bees in large fields with distant forest edges (Lentini et al. 2012). Brosi et al. (2008) proposed a model farm configuration that would maximize crop yield; the highest-yield farm designs were those with a relatively small area of pollination reservoirs, suggesting a conservation strategy of small parcels of service-providing habitat interspersed throughout working landscapes. However, small pollination reservoirs are probably not complete habitats in themselves, so this farm design is likely to be dependent on bee flight range and their ability to disperse throughout the crop matrix (Bommarco et al. 2010). All these results suggest that forest edges are important sources of pollinators because they provide different “partial habitats” for bees (Westrich 1996). However, forest edges need to be spatially well integrated into the agricultural matrix: (i) to promote bee populations, (ii) to ensure pollination services, and (iii) to enhance opportunities for colonization via connecting habitats (Steffan-Dewenter et al. 2002). Unfortunately, trees are often negatively perceived by farmers because they compete with crops for sunlight, nutrients, or water (Huth et al. 2010). Yet, studies show that forest edges or trees may provide several additional ecosystems services such as pest control (Bianchi et al. 2005; Stutz et al. 2011), soil quality improvement, water regulation (Tsonkova et al. 2012), and wind breaks (Brandle et al. 2004). The loss in crop yield induced by forest edges should therefore be weighed up against the potential ecological benefits gained. We recommend that forest edges should be included in agro-environmental schemes and “green belt networks” (http://www.legrenelle-environnement.fr/spip.php, MEEDDM 2010) to promote bee populations, bee biodiversity, and diverse ecosystem services. We also recommend that forest edges should be associated with other agro-environmental schemes, such as fallow land or hedgerows, to supply partial habitats for different bee species throughout the bees' seasonal activity (Hannon and Sisk 2009; Lye et al. 2009).

## Supporting Information

Data S1 Fitted GAMM model of the response of total abundance, richness, mean female ITD, *Andrena* female abundance, *Andrena* male abundance and total *Nomada* abundance as a function of distance from the forest edge for the two qualitative variables “cold” and “warm” orientation.
