# Supplementary material for: Distance from forest edge affects bee pollinators in oilseed rape fields
Source: Ecol Evol. 2014 Jan 15;4(4):370–80. doi: 10.1002/ece3.924 (PMC3936384; doi:10.1002/ece3.924)
Supplement: Data S1 — Fitted GAMM model of the response of total abundance, richness, mean female ITD, Andrena female abundance, Andrena male abundance and total Nomada abundance as a function of distance from the forest edge for the two qualitative variables “cold” and “warm” orientation. [file ece30004-0370-sd1.docx]

Data S1: Fitted GAMM model of the response of total abundance, richness, mean female ITD, *Andrena* female abundance, *Andrena* male abundance and total *Nomada* abundance as a function of distance from the forest edge for the two qualitative variables “cold” and “warm” orientation. We added a regression line through the scatter of data points.

Fitted total abundance


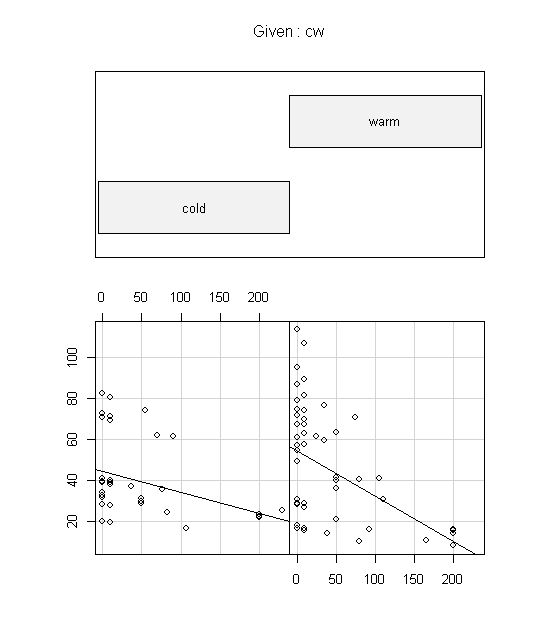

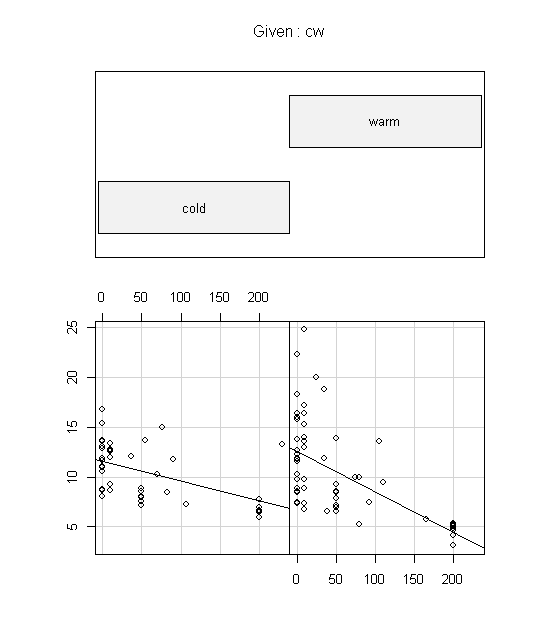


Fitted richness


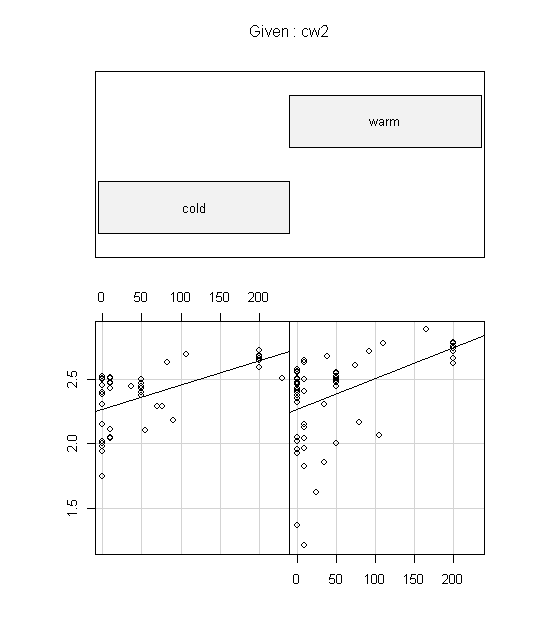


Fitted mean female ITD


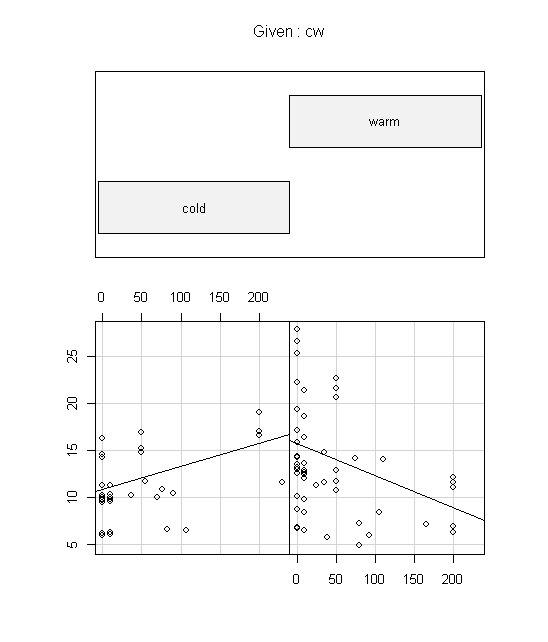


Fitted *Andrena* female abundance


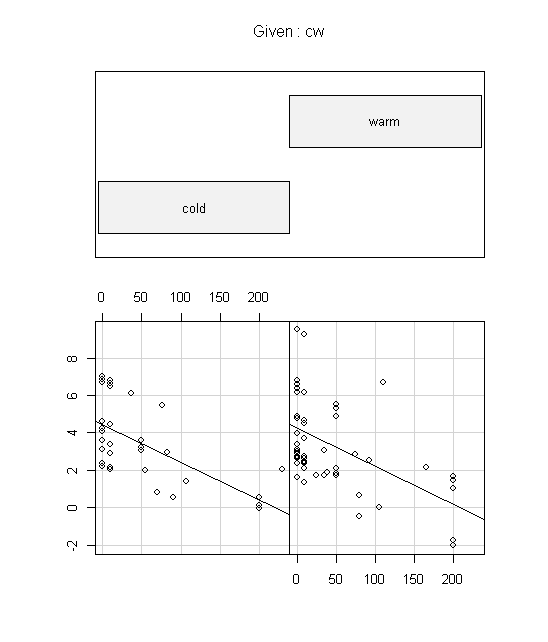


Fitted *Andrena* male abundance


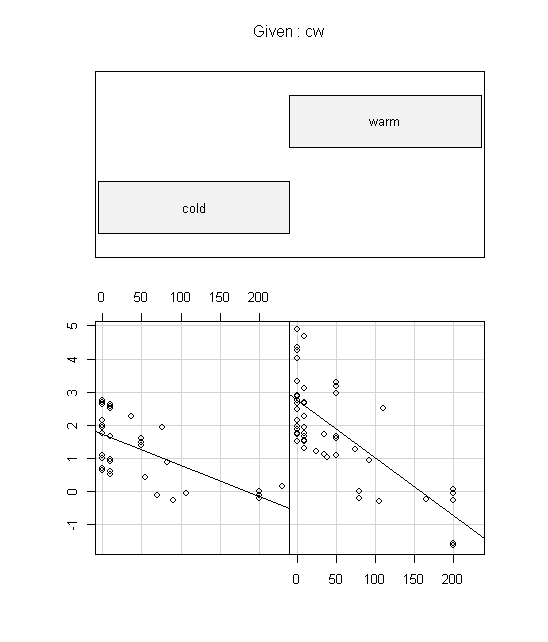


Fitted total *Nomada* abundance

**Cold**

**Warm**

**Cold**

**Warm**

Distance from forest edge in meters

Distance from forest edge in meters
